# Chromosomal Polymorphism in the *Sporothrix schenckii* Complex

**DOI:** 10.1371/journal.pone.0086819

**Published:** 2014-01-23

**Authors:** Alexandre A. Sasaki, Geisa F. Fernandes, Anderson M. Rodrigues, Fábio M. Lima, Marjorie M. Marini, Luciano dos S. Feitosa, Marcus de Melo Teixeira, Maria Sueli Soares Felipe, José Franco da Silveira, Zoilo P. de Camargo

**Affiliations:** 1 Discipline of Cellular Biology, Department of Microbiology, Immunology and Parasitology, Federal University of São Paulo, São Paulo, São Paulo, Brazil; 2 Discipline of Parasitology, Department of Microbiology, Immunology and Parasitology, Federal University of São Paulo, São Paulo, São Paulo, Brazil; 3 Biomedical Engineering Center, Camilo Castelo Branco University, São Paulo, São Paulo, Brazil; 4 Department of Cell Biology, Biological Sciences Institute, University of Brasília, Brasília, Distrito Federal, Brazil; 5 Genomic Sciences and Biotechnology, Catholic University of Brasília, Brasília, Distrito Federal, Brazil; Universidade de Sao Paulo, Brazil

## Abstract

Sporotrichosis is a polymorphic disease caused by a complex of thermodimorphic fungi including *S. brasiliensis, S. schenckii sensu stricto* (*s. str.*), *S. globosa* and *S. luriei.* Humans and animals can acquire the disease through traumatic inoculation of propagules into the subcutaneous tissue. Despite the importance of sporotrichosis as a disease that can take epidemic proportions there are just a few studies dealing with genetic polymorphisms and genomic architecture of these pathogens. The main objective of this study was to investigate chromosomal polymorphisms and genomic organization among different isolates in the *S. schenckii* complex. We used pulsed field gel electrophoresis (PFGE) to separate chromosomal fragments of isolated DNA, followed by probe hybridization. Nine loci (β-tubulin, calmodulin, catalase, chitin synthase 1, Internal Transcribed Spacer, Pho85 cyclin-dependent kinase, protein kinase C Ss-2, G protein α subunit and topoisomerase II) were mapped onto chromosomal bands of Brazilian isolates of *S. schenckii s. str.* and *S. brasiliensis*. Our results revealed the presence of intra and interspecies polymorphisms in chromosome number and size. The gene hybridization analysis showed that closely related species in phylogenetic analysis had similar genetic organizations, mostly due to identification of synteny groups in chromosomal bands of similar sizes. Our results bring new insights into the genetic diversity and genome organization among pathogenic species in the *Sporothrix schenckii* complex.

## Introduction

Sporotrichosis is a chronic, granulomatous, subcutaneous disease caused by several thermodimorphic pathogenic species in the *Sporothrix schenckii* complex [1]. It is widely reported that the infection occurs through traumatic inoculation of fungal propagules into the skin by contaminated material, such as soil, thorns or splinters. Sporotrichosis mainly affects humans and animals [2]–[4]. The zoonotic potential, as exemplified by animal scratches and bites, particularly from cats, are the most common modes of transmission to humans in hyperendemic areas in Brazil [5], [6]. In some cases, human infections are associated with transmission from wild animals; for example, injuries caused by armadillos when hunting the animal [7]. Sporotrichosis has a worldwide distribution with a high incidence in temperate and tropical regions including Latin America (Brazil, Mexico, Colombia, Costa Rica, Guatemala, and Uruguay), South Africa, India, and Japan [8].

Currently, medically relevant *Sporothrix* spp. in the *S. schenckii* complex are *S. brasiliensis* (Clade I), *S. schenckii sensu stricto* (*s. str.*) (Clade II), *S. globosa* (Clade III), and *S. luriei* (Clade VI) [1]; while, *S. mexicana* (Clade IV) and *S. pallida* (Clade V) are remotely related in phylogenetic analysis [1], [3], [6] and, therefore, are considered apart from the clinical group. These species were identified based on multi-locus sequencing, morphology, and physiological characteristics [9], [10]. *Sporothrix schenckii s. str.* is found predominantly on the American, Asian, and African continents; *S. globosa* is a widespread species, found with high frequency in Europe and Asia; and *S. brasiliensis* has been isolated exclusively in Brazil [1], [9]. *Sporothrix luriei* is a rare pathogen. Four human cases have been reported, but the etiological agent was only isolated in the first reported case [11]. In Brazil, over the last decades, the incidence of sporotrichosis has increased exponentially to epidemic proportions. The epidemic occurred mainly in Rio de Janeiro, and was caused by *S. brasiliensis* [6], [9].

Recent studies have shown important differences in virulence and drug resistance profiles among different species in the *S. schenckii* complex. *Sporothrix brasiliensis* is the most virulent species. In contrast, *S. globosa* and *S. mexicana* showed little or no virulence in a murine model [12]–[14]. When compared for drug resistance, *S. brasiliensis* was less resistant than other species, and *S. mexicana* was the most resistant [15]. Despite these findings, little is known about the genetic mechanisms of virulence and drug resistance in the *S. schenckii* complex.

Fungi present complex genomes and fungal chromosomes are small and difficult to visualize with traditional karyotyping techniques [16]. The development of pulsed field gel electrophoresis (PFGE) by Schwartz and Cantor [17] and improvements of this technique over the years have promoted a novel method for studying fungal chromosomes. With this method, the molecular karyotypes of eight *S. schenckii sensu lato* (*s.l.*) isolates were defined in Japan. Those isolates appeared to include 6 to 8 chromosomal bands, and each band contained one or more chromosomes of the same size. The genome size was estimated to be approximately 28 Mb [18]. In that study, the authors applied four distinct conditions to separate and estimate the chromosome numbers and sizes for *S. schenckii s.l.* isolates. The four different conditions used made it difficult to compare similar chromosomes, and that approach may not have provided accurate estimates of the genome size. Furthermore, that study was performed before the new *Sporothrix* species had been defined; hence, it is not clear whether the authors studied *S. schenckii s. str.* or isolates of *S. globosa*, which are also found in the Asian territory.

The genomic organization and chromosome number in different species in the *S. schenckii* complex remain unknown. The aim of the present study was to investigate chromosomal polymorphisms among *Sporothrix* spp. isolates from diverse geographic origins with the PFGE technique. Additionally, we used hybridization to map the location of nine genetic markers onto the chromosomal bands of Brazilian isolates of *S. schenckii s. str.* and *S. brasiliensis*. Our results showed the existence of polymorphisms in the number and size of chromosomes among different *Sporothrix* isolates. This information will be useful for genetic mapping and for future studies on the chromosomal organization and epidemiology of the *S. schenckii* complex.

## Materials and Methods

### Fungal Strains and Growth Conditions

Fungal isolates (n = 23) were grown on Sabouraud agar slants at room temperature for 7 days. Then, the total growth from each slant was transferred to a 500 mL Erlenmeyer flask containing 50 ml of Brain Heart Infusion (BHI) broth (Acumedia, USA), supplemented with dextrose (18 g/L), pH 8.0, and incubated at 37°C for 10 days, under gentle shaking. This procedure induced yeast formation. The codes and information for all isolates are summarized in Table 1.

**Table 1:** Strains, species, origin, and GenBank accession numbers (CAL and ITS) for the *Sporothrix* spp. isolates used in this study.

Isolate	Other Code	Species	Source	Country	CAL	ITS	Reference	
IPEC 16919	FMR 8314, Ss177	*S. brasiliensis*	Human	Brazil		AM116899	KF574441	9,10, This study
CBS 120339^T^	IPEC 16490, Ss178	*S. brasiliensis*	Human	Brazil		AM116898	KF574440	9, 10, This study
ATCC MYA-4823	CBS 132021, Ss322	*S. brasiliensis*	Feline	Brazil		KF574459	JQ070114	This study
Ss99	–	*S. brasiliensis*	Human	Brazil		KF574460	KF574442	This study
Ss104	–	*S. brasiliensis*	Human	Brazil		KF574461	KF574443	This study
Ss52	–	*S. brasiliensis*	Human	Brazil		KC693845	KF574444	6, This study
Ss54	CBS 132990	*S. brasiliensis*	Feline	Brazil		JQ041903	JN885580	3, 14
CBS 130106	IPEC 15572	*S. brasiliensis*	Human	Brazil		AM116886	FN549903	1, 9, 10, 32
CBS 130108	IPEC 17943	*S. brasiliensis*	Human	Brazil		AM116878	FN549902	1, 9, 10, 32
Ss265	CBS 133020	*S. brasiliensis*	Human	Brazil		JN204360	KF574445	34, 6, This study
CBS 130109	IPEC 22582	*S. brasiliensis*	Human	Brazil		AM116891	KC113213	1, 9
CBS 130107	IPEC 17521	*S. brasiliensis*	Human	Brazil		AM116874	KC113214	1, 9
CBS 359.36^T^	Ss185	*S. schenckii*	Human	USA		AM117437	FJ545232	9, 10, 32
Ss03	CBS 132963	*S. schenckii*	Human	Brazil		JX077117	KF574446	3, This study
Ss39	–	*S. schenckii*	Human	Brazil		JQ041899	JN885576	6, 14
Ss51	–	*S. schenckii*	Human	Brazil		JQ041902	JN885579	14
Ss61	–	*S. schenckii*	Environmental	Brazil		KF561244	KF574447	This study
Ss126	–	*S. schenckii*	Human	Brazil		JQ041904	JN885581	14
Ss137	–	*S. schenckii*	Human	Brazil		KF574462	KF574448	This study
Ss140	–	*S. schenckii*	Human	Brazil		KF574463	KF574449	This study
Ss141	CBS132975	*S. schenckii*	Human	Brazil		JQ041905	JN885582	14
Ss159	CBS 132976	*S. schenckii*	Human	Japan		KF574464	KF574450	This study
Ss160	–	*S. schenckii*	Human	Mexico		KF574465	KF574451	This study
Ss161	–	*S. schenckii*	Human	Mexico		KF574466	KF574452	This study
Ss162	CBS 132977	*S. schenckii*	Environmental	Mexico		KF574467	KF574453	This study
Ss163	–	*S. schenckii*	Human	Peru		KF574468	KF574454	This study
Ss164	–	*S. schenckii*	Human	Peru		KF574469	KF574455	This study
ATCC MYA-4821	CBS 132984, Ss323	*S. schenckii*	Human	USA		KF574470	JQ070112	This study
Ss40	–	*S. schenckii*	Human	Brazil		JQ041900	JN885577	14
Ss143	–	*S. schenckii*	Human	Brazil		JQ041906	JN885583	6, 14
CBS 120340^T^	FMR 8600	*S. globosa*	Human	Spain		AM116908	FN549905	9, 10, 32
Ss06	CBS 132922	*S. globosa*	Human	Brazil		JF811336	JN885574	3, 6
Ss41	CBS 132923	*S. globosa*	Human	Brazil		JF811337	KF574456	3, 6, This study
CBS 130116	FMR 8598	*S. globosa*	Human	Spain		AM116903	KC113226	9, 10, 1
CBS 130104	FMR 8595	*S. globosa*	Human	Spain		AM116905	KC113225	9, 10, 1
CBS 130105	FMR 8597	*S. globosa*	Human	Spain		AM116907	FN549904	9, 10, 32
CBS130117	FMR 9022	*S. globosa*	Human	Japan		AM398995	KC113229	9, 1
CBS 130115	FMR 8596	*S. globosa*	Human	Spain		AM116902	KC113228	9, 10, 1
CBS 937.72^T^	ATCC 18616, Ss187	*S. luriei*	Human	South Africa		AM747302	AB128012	11
CBS 120341^T^	FMR 9108, Ss182	*S. mexicana*	Environmental	Mexico		AM398393	FN549906	9, 32
Ss132	CBS 132927	*S. mexicana*	Human	Brazil		JF811340	KF574457	3, 6
Ss327	PG3	*S. pallida*	Feline	Brazil		KF574471	KF574458	This study
CBS 124561^T^	FMR 9338	*S. brunneoviolacea*	Environmental	Spain		KF574472	FN546959	32

IPEC, Instituto de Pesquisa Clínica Evandro Chagas, Fiocruz, Brazil; FMR, Facultat de Medicina i Ciències de la Salut, Reus, Spain; CBS, CentraalBureau voor Schimmelcultures, Utrecht, The Netherlands; ATCC, American Type Culture Collection, Manassas, VA, USA; T, type strain. All “Ss” strains belong to the culture collection of the Federal University of São Paulo (UNIFESP).

### Isolation of Genomic DNA and Preparation of Intact Chromosomal DNA from *Sporothrix* spp. Cells

Genomic DNA was extracted according to the protocol of de Bievre et al. [19], with some modifications. Briefly, yeast cells were washed 3 times with wash buffer (50 mM EDTA, pH 8.0); then, cells were resuspended in protoplast buffer, which contained 5 mM EDTA, 100 mM Tris-HCl pH 7.5, 0.01% (v/v) β-mercaptoethanol, and 0.1 mg/mL zymolyase-20T (MP Biomedicals, USA). Cells were incubated at 37°C for 1 h. Next, lysis buffer was added, which contained 10 mM Tris-HCl, 1 mM EDTA, 1 mg/mL proteinase K, 5% (w/v), and sarkosyl. Cells were incubated for 1 h at 56°C. After centrifugation, the supernatant was collected and mixed with phenol:chloroform:isoamilic alcohol (25∶24∶1) and incubated at room temperature, under gentle shaking, for 10 min. The samples were centrifuged, and supernatants were incubated with absolute ethanol and 300 mM sodium acetate (pH 5.2) at −20°C for 16 h. After centrifugation, the pellet was washed with 70% ethanol dried, and resuspended in TE buffer (10 mM Tris-HCl, 1 mM EDTA, pH 8.0), supplemented with 1 µg/mL RNAse. The solution was incubated for 1 h at 37°C. The remaining DNA in solution was washed with phenol:chloroform:isoamilic alcohol (25∶24∶1) and centrifuged. The water phase was collected, isopropyl alcohol was added, and the suspension was incubated for 16 h at −20°C. Finally, the samples were centrifuged, and the pellet washed with 70% ethanol. The pellet was allowed to dry at 37°C, then resuspended in sterile water, and stored at −20°C until use.

We embedded cells within agarose plugs that could be inserted into a PFGE system for chromosome isolation. PFGE plugs were prepared according to the protocol of Herschleb et al. [20], with minor modifications. Briefly, yeast cells were harvested from BHI broth and washed 3 times with wash buffer (EDTA 50 mM, pH 8.0); 2×10^8^ yeast cells were used to prepare each agarose plug. The cells were mixed with Zymolyase solution (1 mg/mL Zymolyase-20T in glycerol-phosphate buffer, pH 7.0) and 1% Low Melting Point Agarose (FMC BioProducts, USA) to achieve a final concentration of 1×10^8^ cells/mL. Then, the cell suspension was pipetted into a casting mold, and the agar was allowed to solidify at 4°C. The plugs were removed from the molds, and a spheroplast solution (0.5 M EDTA, 7.5% β-mercaptoethanol, pH 8.0) was added. The PFGE plugs were incubated overnight at 37°C. The spheroplast solution was replaced with NDSK buffer, which contained 0.5 M EDTA, 1% (w/v) *N*-laurylsarcosine (Sigma, USA), and 1 mg/ml Proteinase K (Sigma, USA), pH 9.5. Then, the PFGE plugs were washed 3 times with wash buffer, and incubated overnight at 50°C. After washing 3 times with wash buffer, the NDSK buffer was replaced with stock solution (0.5 M EDTA, pH 9.5) and stored at 4°C until use.

### Pulsed Field Gel Electrophoresis

The PFGE separation was performed according to Feitosa et al. [21], with minor modifications. Briefly, DNA plugs were loaded onto the PFGE gel (0.8%), and chromosomal separation was carried out in the Gene Navigator System (Amersham Pharmacia Biotech, USA) at a constant temperature of 10°C. The best chromosomal band resolution was achieved in 1× TAE buffer with homogeneous pulses and interpolation for 168 h at 42 V (phase 1: pulse time 900 s/24 h; phase 2∶1800 s/24 h; phase 3∶2700 s/48 h; phase 4∶3600 s/48 h; phase 5∶4500 s/24 h). *Schizosaccharomyces pombe* chromosomes (Bio-Rad, USA) were run in parallel as standard size markers. After electrophoresis, the gel was stained with 0.5 µL/mL ethidium bromide (0.5 µL/mL; EtBr) for 15 min, and washed with distilled water for 15 min. The digital images of the gel were acquired with a L-Pix Touch documentation system (Loccus Biotecnologia, Brazil).

### Densitometric Analysis

Densitometric analysis was performed with LabImage 1D, ver. 3.3.4 software (Kapelan Bio-Imaging, Germany), and chromosomal band sizes were estimated. Each chromosomal band size was calculated by comparing its migration with that of *S. pombe* chromosomes, which were used as standard size markers.

### PCR Amplification and DNA Probes

PCR was used to amplify gene fragments from β-tubulin (β-tub) [10], calmodulin (Calm) [22], catalase (Cata) [23], chitin synthase 1 (CHS1) [24], Internal Transcribed Spacer (ITS) [25], Pho85 cyclin-dependent kinase (Pho85) [26], protein kinase C Ss-2 (PKC) [27], G protein α subunit (GProt) [28], and topoisomerase II (Topo) [29] with primers designed to target specific gene sequences (Table S1). The DNA sample from *S. schenckii s. str.* (Ss126) (100 ng/µL) was also amplified in PCR reactions with the PCR Master Mix (Promega, USA), according to the manufacturer’s protocols. Each amplicon was cloned into the pGEM-T Easy Vector System (Promega, USA), following the manufacturer’s instructions. These plasmids were transfected into Subcloning Efficiency DH5-α Competent Cells (Invitrogen, USA) according to the manufacturer’s protocol. Plasmids were purified with the NucleoSpin Plasmid kit (Macherey-Nagel, Germany) and sequenced with the BigDye Terminator v3.1 Cycle Sequencing Kit (Applied Biosystems, USA) and an ABI 3730 DNA Analyzer (Applied Biosystems, USA).

### Radiolabeling Probes and Southern Blot Hybridization

Specific probe sequences were prepared by excising DNA fragments from the plasmids with the *Eco*RI restriction enzyme (Fermentas, USA), according to the manufacturer’s instructions. The probes were purified with the NucleoSpin Extract II kit (Macherey-Nagel, Germany). In addition, the entire 7.0 Mb chromosomal band of isolate Ss126 was excised from an agarose gel, and the agarose was digested with β-agarase (Lonza, USA), according to the manufacturer’s protocol. Also, genomic DNA was digested with the *Hae*III restriction enzyme (Fermentas, USA), according to the manufacturer’s instructions, then the fragments were purified to use as a probe. All probes were radiolabeled with the Random Primers Labeling System kit (Invitrogen, USA) and dCTP (α-P32) (Perkin Elmer, USA).

After PFGE, chromosomal bands were transferred to a Hybond-N nylon membrane (Amersham Bioscience, United Kingdom) by capillary action at room temperature. DNA was fixed to the membrane in a GS Gene Linker UV™ Chamber (Bio-Rad, USA) under UV irradiation (150 mJ). The fixed membranes were stored at −4°C until use.

For hybridization, the membranes were incubated with pre-hybridization buffer, which contained 0.25 M Na_2_PO_4_, 2 mL phosphoric acid (85%), 7% SDS, 1% BSA, and 1 mM EDTA, for 30 min at 65°C. The radiolabeled probes were added, and hybridization was carried out at 65°C, for 16 h. The membranes were washed once in 20 ml of Solution I (0.25 M Na_2_PO_4_, 2 mL phosphoric acid, 1% SDS, and 1 mM EDTA) at 65°C for 30 min, and then once in 20 ml of solution II (0.125 M Na_2_PO_4_, 1 mL phosphoric acid [85%], 1% SDS, and 1 mM EDTA) for 30 min. Next, the membranes were exposed to x-ray film (GE Healthcare, United Kingdom) for 2 to 4 days at −80°C, and finally, the film was developed.

### Phylogenetic Analysis

The calmodulin gene [9] and ITS region fragments [1] were amplified directly from genomic DNA with primer sets CL1 and CL2A (for calmodulin) and ITS1 and ITS4 (for ITS). Amplified products were gel-purified with the Wizard SV Gel and PCR Clean-Up System (Promega Corp., USA), according to the manufacturer’s instructions. DNA samples were sequenced as described above. Fragments were sequenced on both strands to increase the quality of sequence data (Pherd>30). Alignment of sequences was performed with the ClustalW algorithm [30], implemented in BioEdit software [31]. Retrieved alignments were manually corrected to avoid mispaired bases. Nucleotide sequences were exported into a FASTA file to use in a BLAST search (www.ncbi.nlm.nih.gov/BLAST).

For reference sequences, this study included calmodulin sequences (n = 43) from other isolates of *S. schenckii s. str.* (n = 8), *S. brasiliensis* (n = 9), *S. globosa* (n = 8), and *S. mexicana* (n = 2), and ITS sequences (n = 43) from isolates of *S. schenckii s. str.* (n = 8), *S. brasiliensis* (n = 6), *S. globosa* (n = 7), *S. mexicana* (n = 1), and *S. brunneoviolacea* (n = 1). These sequences were previously deposited in GenBank (www.ncbi.nlm.nih.gov/genbank) and described by Marimon et al. [9], [10], Rodrigues et al. [3], [6], Fernandes et al. [14], Madrid et al. [32], Zhou et al. [1], Oliveira et al. [33], and Silva-Vergara et al. [34] (Table 1). The calmodulin and ITS sequences were concatenated with FaBox software [35], available at http://www.birc.au.dk/fabox. Evolutionary analysis was performed in MEGA5 [36] with Neighbor-joining and Maximum Likelihood methods. To estimate confidence values for individual clades, the Tamura 3-parameter nucleotide substitution model was used [37], with a gamma distribution (shape parameter = 1) rate variation among sites, and 1,000 bootstrap replicates [38].

### Ethics Statement

The procedures performed in this study were approved by Research Ethics Committee of UNIFESP [CEP N°. 0111/11].

## Results

### Phylogenetic Analysis

**Figure 1:**
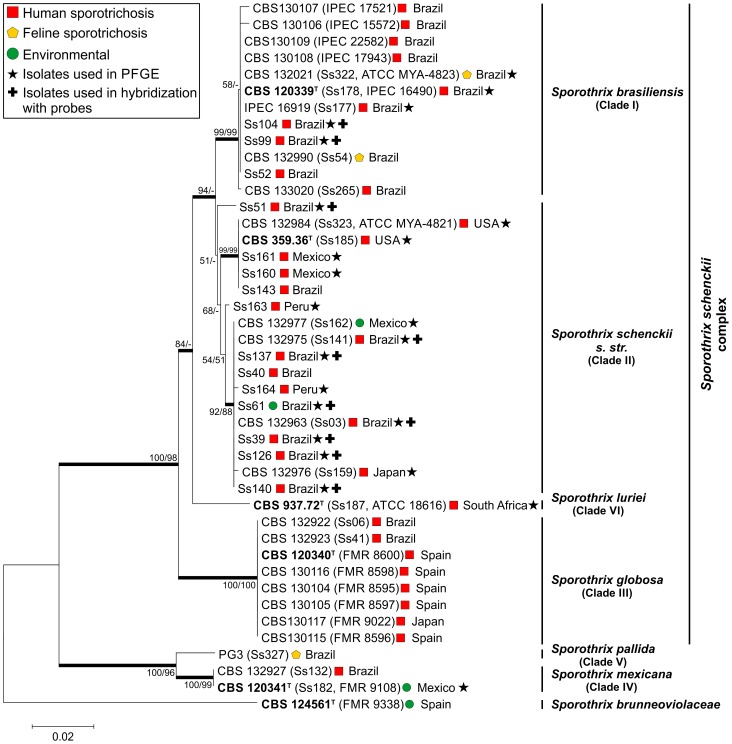
Molecular phylogenetic tree generated by Neighbor Joining and Maximum Likelihood methods with the concatenated sequences of calmodulin and internal transcribed spacer (ITS). In the bootstrap test (1,000 replicates), replicate trees in associated taxa were clustered together. The bootatrap for each tree branch is shown next to the corresponding branch. Star symbols indicate that the isolate was used for pulsed field gel electrophoresis (PFGE). The cross symbols indicate isolates used in hybridization experiments with genetic markers (probes).

The Calm and ITS regions were amplified with PCR, which yielded fragments of approximately 800 bp and 620 bp, respectively. These sequences were aligned and concatenated for phylogenetic analysis. The concatenated sequences were 1,389 bp long and included 872 invariable characters (63%), 224 variable parsimony-informative characters (16%), and 183 singleton characters. Positions that contained gaps or missing data were eliminated. The 43 concatenated sequences were clustered into 7 main groups. Clades I–III and VI (*S. brasiliensis*, *S. schenckii s. str.*, *S. globosa* and *S. luriei*, respectively) are related to human and animal infections, while *S. pallida* (Clade V), *S. mexicana* (Clade IV) and *S. brunneoviolacea* are well described as soil and decaying wood inhabiting (Fig. 1).

In general, *S. schenckii* (Clade II) isolates were highly diverse; most isolates from Brazil preferentially clustered into the subclade opposite to the type strain (CBS 359.36). However, the isolates from Mexico and Peru were distributed through the clade II. On the other hand, clade I (*S. brasiliensis*) and clade III (*S. globosa*) appeared to be more homogenous, with low genetic diversity.

### Molecular Karyotype and Chromosomal Polymorphisms in the *S. schenckii* Complex

The chromosomal bands of *Sporothrix* spp. isolates were separated by PFGE and stained with EtBr. A visible chromosomal band on the PFGE may contain one or more co-migrating chromosomes which are not necessarily homologous.

**Figure 2:**
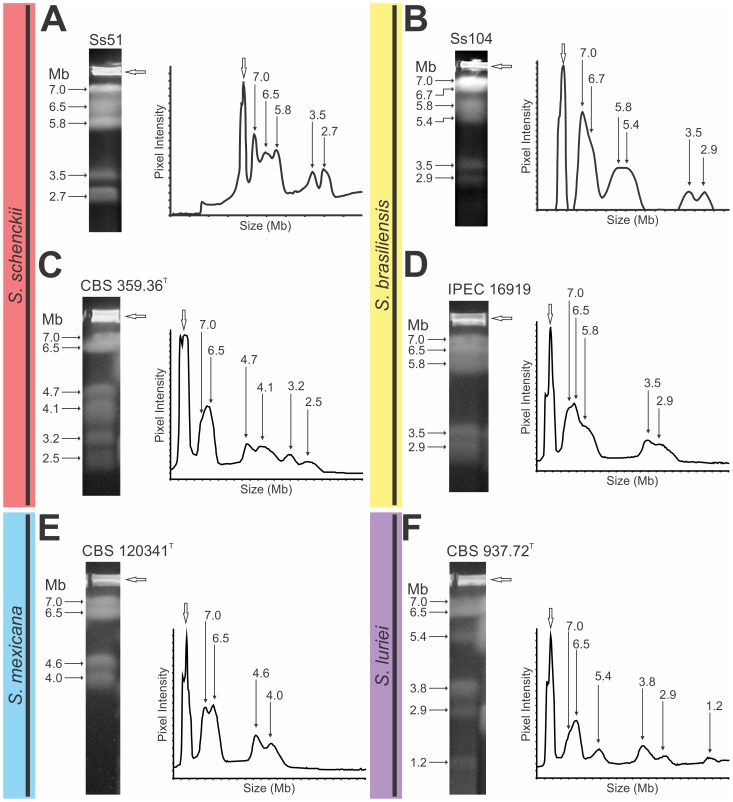
Densitometric analysis of *S. schenckii*, *S. brasiliensis*, *S. mexicana*, and *S. luriei* isolates. Each panel shows (*left*) the ethidium bromide-stained gel after pulsed field gel electrophoresis of chromosomes from the fungus strain indicated, and (*right*) a graph of the densitometric analysis. The size of each chromosomal band (Mb) is indicated on the left and above the corresponding peaks on the graph. Open arrows indicate where samples were loaded.

In the densitometric analysis of chromosomal bands separated with PFGE, we determined the numbers and sizes of chromosomes among 16 isolates of the *S. schenckii* complex. The different *S. schenckii s. str.* isolates displayed remarkable differences in chromosome profiles. The isolates had 4 to 7 chromosomal bands, ranging from 2.0 to 7.0 Mb (Fig. 2A, C; Figs. S1 and S2). In contrast, *S. brasiliensis* isolates showed fewer polymorphisms; in which the karyotype was composed of 5 to 7 chromosomal bands, ranging from 2.9 to 7.0 Mb (Fig. 2B, D; Fig. S3). Interestingly, several isolates of *S. schenckii s. str.* and *S. brasiliensis* had similar electrophoretic karyotypic profiles (Fig. 2A and Fig. S1A–C; Fig. 2D and Fig. S3A, C, respectively). The isolates of *S. mexicana* and *S. luriei* had karyotype profiles composed of 4 (ranging from 4.0 to 7.0 Mb) and 6 (ranging from 1.2 to 7.0 Mb) chromosomal bands, respectively (Fig. 2E–F).

**Figure 3:**
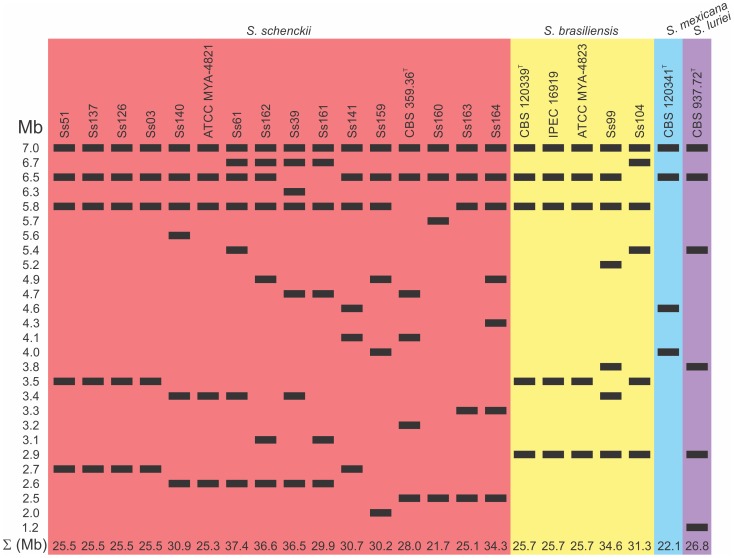
Schematic representation of chromosomal bands of *Sporothrix spp.* isolates. Each species is grouped inside a colored rectangle. Red: *S. schenckii*; Yellow: *S. brasiliensis*; Blue: *S. mexicana*; Purple: *S. luriei*. The sizes of chromosomal bands (Mb) are shown on the left. Σ: The minimum genome sizes estimated by summing the chromosomal bands (Mb) are shown at the bottom.

To facilitate visualization and comparisons among the karyotype profiles of all isolates from the *S. schenckii* complex, we created a schematic of chromosomal bands (Fig. 3). Most chromosomal bands of *S. schenckii s. str.* and *S. brasiliensis* isolates ranged in size from 7.0 to 5.8 Mb and from 3.5 to 2.5 Mb. Several isolates also had bands ranging from 5.7 to 3.8 Mb. *Sporothrix mexicana* had no chromosomal bands smaller than 4.0 Mb, but the *S. luriei* isolate had a small chromosomal band of 1.2 Mb. The electrophoretic karyotypes were not correlated with the source of isolation (i.e., human or environment) or with the clinical characteristics of the disease.

### Mapping *S. schenckii s. str.* Genes in Chromosomal Bands of Different Isolates

**Figure 4:**
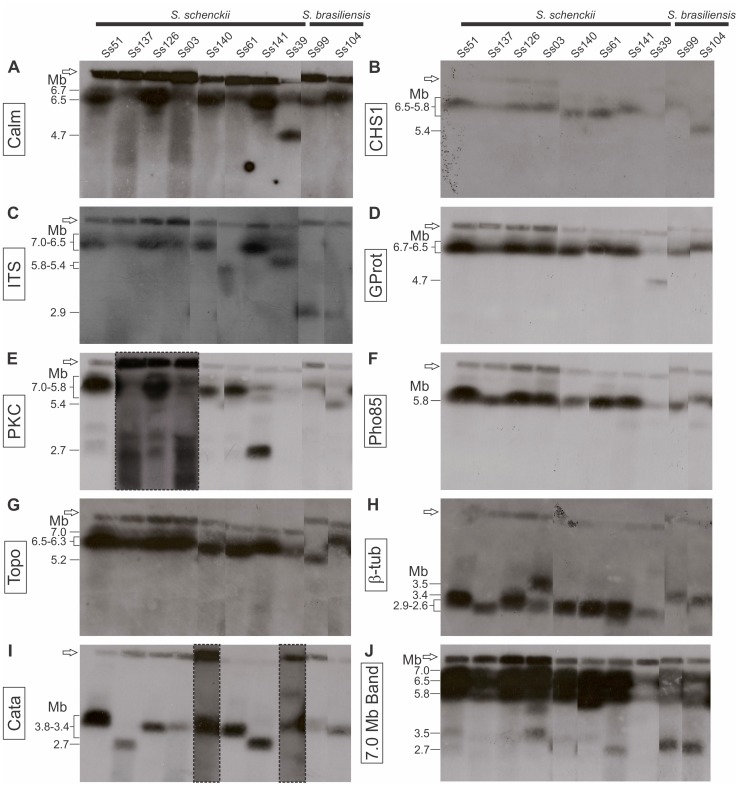
Specific gene probes hybridized to chromosomal bands of Brazilian isolates of *S. schenckii* and *S. brasiliensis*. Southern blots show radiolabeled probes hybridized to fungi chromosomes. Probes that specifically targeted A: calmodulin (Calm), B: chitin synthase 1 (CHS1), C: Internal Transcribed Spacer (ITS), D: G protein α subunit (GProt), E: protein kinase C Ss-2 (PKC), F: Pho85 cyclin-dependent kinase (Pho85), and G: topoisomerase II (Topo) hybridized preferentially to large bands in most isolates. Probes for H: β-tubulin (β-tub) and I: catalase (Cata) hybridized preferentially to small bands in most isolates. J: The 7.0 Mb chromosomal band from isolate Ss126 was used as a probe. The hybridization pattern shows evidence for repetitive sequences. The sizes of major chromosomal bands (Mb) are indicated on the left. The open arrows indicate the wells where samples were loaded. Areas enclosed in a dashed box (E and I) are lanes that were exposed for longer times to better visualize the hybridization signals.

Due to the high prevalence of *S. brasiliensis* and *S. schenckii s. str.* in Brazil [3], [6], [9], we evaluated the differences in genome structures among eight *S. schenckii s. str.* isolates and two *S. brasiliensis* isolates collected in distinct regions of Brazil. The hybridization profiles generated with nine genetic markers were very similar among the *S. schenckii s. str.* and *S. brasiliensis* isolates. Some probes, like Calm, CHS1, ITS, GProt, PKC, Pho85, and Topo, hybridized preferentially with the large chromosomal bands (Fig. 4A–G). On the other hand, other probes, like β-tub and Cata, hybridized preferentially with small chromosomal bands (Fig. 4H–I).

**Figure 5:**
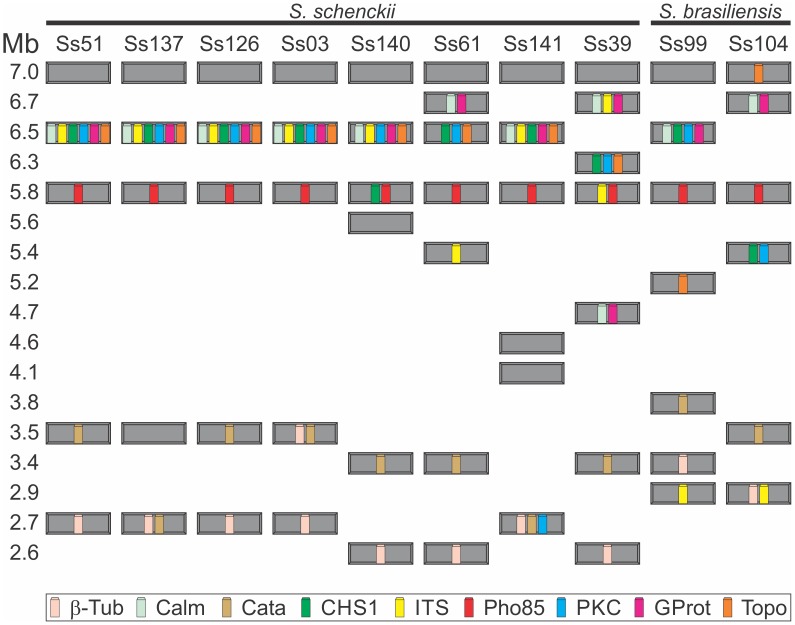
Schematic of probe hybridization to fungal chromosome bands. The *S. schenckii* and *S. brasiliensis* isolates are indicated at the top. Each grey box represents a chromosomal band with the indicated size (Mb, *left*). The probes that hybridized to each band are color-coded (*bottom*). β-tubulin (β-Tub), calmodulin (Calm), catalase (Cata), chitin synthase 1 (CHS1), internal transcribed spacer (ITS), Pho85 cyclin-dependent kinase (Pho85), protein kinase C Ss-2 (PKC), G protein α subunit (GProt), and topoisomerase II (Topo).

The probe hybridization patterns on chromosomal bands are shown schematically in Figure 5. The 6.5 Mb chromosomal band hybridized with 3 to 6 probes. Four isolates of *S. schenckii* showed a similar pattern of hybridization (Ss51, Ss137, Ss126 and Ss03) on the 6.5 Mb chromosomal band; in figure 5 the colors indicate the six different probes that hybridized with this band: Calm, CHS1, ITS, PCK, GProt, and Topo. Interestingly, probe Pho85 hybridized with the 5.8 Mb chromosomal band in all isolates. The ITS probe hybridized with small chromosomal bands in *S. brasiliensis* isolates, but with large chromosomal bands in *S. schenckii s. str*. In addition to this general observation, it is interesting to note that some chromosomal polymorphisms were detected, mainly in the *S. schenckii s. str.* isolates. We identified several possible events, including translocation and duplication.

Mapping the chromosomal bands with the catalase probe yielded weak hybridization signals in isolates Ss140 and Ss39. Those signals could be clarified when the Hybond nylon membrane was subjected to longer exposure times. The same occurred with PKC in isolates Ss137, Ss126, and Ss03 (Fig. 4E, I). We also observed that Calm, GProt, and ITS probes hybridized with two chromosomal bands in the Ss39 isolate; Calm and GProt hybridized with the 6.7 Mb and 4.7 Mb bands, and ITS hybridized with the 6.7 Mb and 5.8 Mb bands. This suggested the possibility that a gene duplication event may have occurred in this isolate (Fig. 5). In addition, a comparison of the hybridization profiles showed that Calm and GProt were present in all isolates. It is tempting to suggest that those two genes may constitute a linkage group; therefore, they may be two parts of the same DNA molecule. Another possible gene duplication event occurred in the Ss03 isolate; this involved the β-tubulin gene which hybridized to two chromosomal bands of 3.5 Mb and 2.7 Mb. Whether only the gene locus or the entire chromosome was duplicated remains to be demonstrated.

We also identified a possible translocation event of PKC gene. In Ss141 isolate, the PKC probe strongly hybridized to the 2.7 Mb chromosomal band while in others isolates it hybridized on larger size bands (6.5 Mb, 6.3 Mb and 5.4 Mb). Other genes (CHS1, Topo, GProt and Calm) that were mapped with PKC on larger size bands did not move to 2.7 Mb band (Fig. 4), supporting the hypothesis above. Despite the previously reported [39] diploid nature of *S. schenckii s.l.*, our results suggested that this organism may be aneuploid for several chromosomes.

To establish a correlation among the 7.0 Mb chromosomal bands observed in all isolates, this band from isolate Ss126 was excised from the gel, radiolabeled with ^32^P, and hybridized to the chromoblots. The 7.0 Mb probe hybridized preferentially with the large chromosomal bands in *S. schenckii* isolates. This hybridization pattern suggested the presence of repetitive sequences in *S. schenckii s. str.,* particularly in the large chromosomal bands (Fig. 4J). The weak hybridization signals in isolate Ss39 may have been due to low amounts of DNA in the PFGE plugs, because the plugs prepared with this isolate appeared to yield less DNA than that yielded with the other isolates. In *S. brasiliensis* isolates, the hybridization signal was less intense for the larger chromosomal bands and more intense for the smaller bands (Fig. 4J). The hybridization intensity was different between the two *Sporothrix* species, with a stronger signal in *S. schenckii s. str.* than in *S. brasiliensis*. This may have reflected a difference in the content of repetitive sequences.

### Estimation of Genome Size

**Figure 6:**
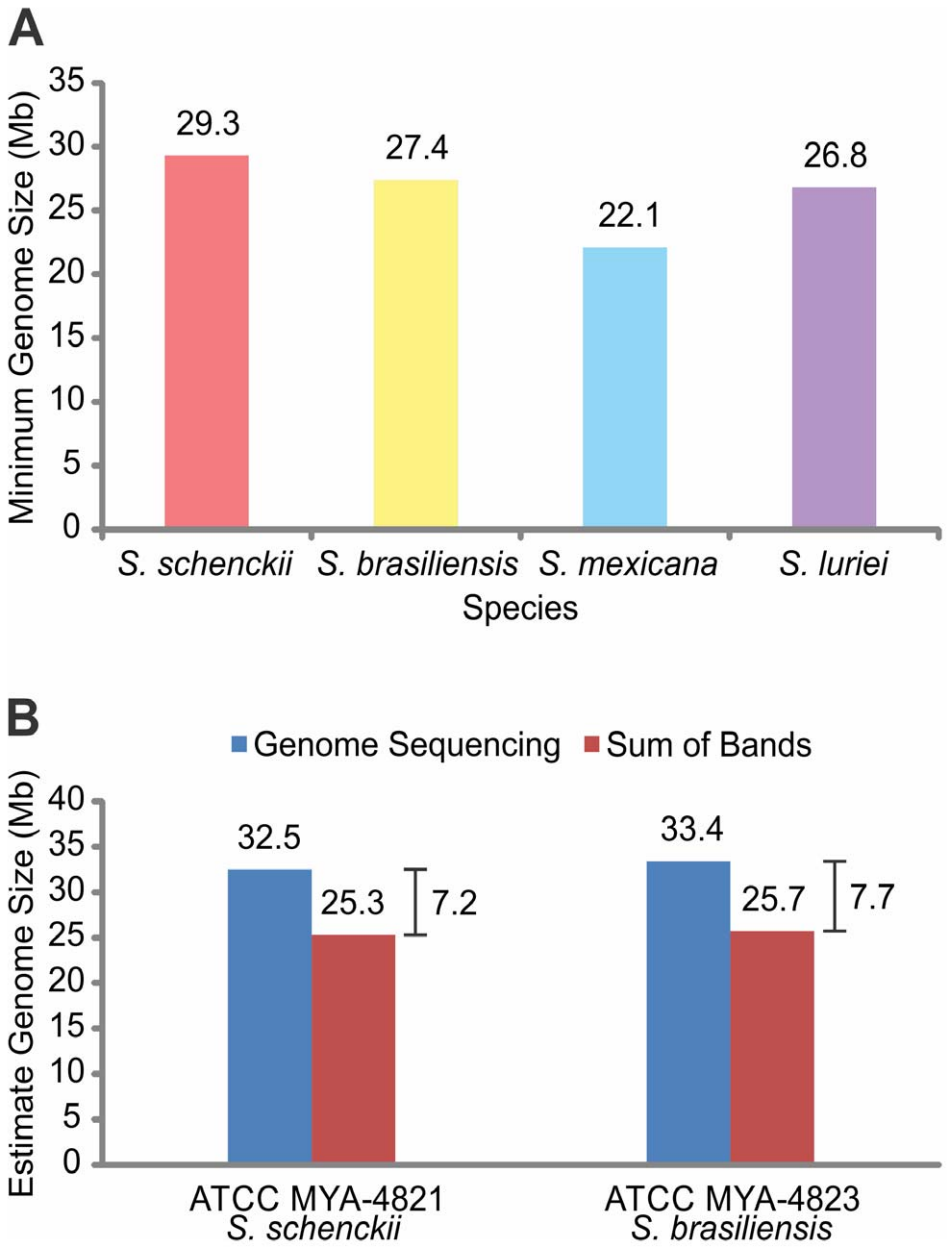
Estimation of genome sizes for *Sporothrix* isolates. A: The average minimum genome size for different *Sporothrix* species was estimated by summing all the chromosomal bands detected after pulsed field gel electrophoresis. B: Comparison of genome sizes estimated from genome sequencing (blue) and estimated from the sum of chromosomal bands (red) for isolates ATCC MYA-4821 (*S. schenckii*) and ATCC MYA-4823 (*S. brasiliensis*); the differences in size estimated with the different methodologies are indicated on the right.

The minimum size of a genome was estimated as the sum of the individual chromosomal bands. The minimum genome size of *S. schenckii s. str.* isolates varied from 25.1 to 37.5 Mb, and that of *S. brasiliensis* isolates varied from 25.7 to 34.7 Mb (Fig. 3). The average genome sizes of *S. schenckii s. str.* and *S. brasiliensis* were 29.3 Mb and 27.4 Mb, respectively; the genome sizes of *S. mexicana* and *S. luriei* isolates were 22.1 Mb and 26.8 Mb, respectively (Fig. 6A).

Based on genomic data from the genome sequencing project of *S. schenckii s. str.* (ATCC MYA-4821) and *S. brasiliensis* (ATCC MYA-4823) isolates, the genome sizes of ATCC MYA-4821 and ATCC MYA-4823 were estimated to be 32.5 Mb and 33.4 Mb, respectively (Marcus de Melo Teixeira and Maria Sueli Soares Felipe, personal communication). In the present study, the minimum genome sizes evaluated by PFGE of these isolates were estimated to be 25.3 Mb (ATCC MYA-4821) and 25.7 Mb (ATCC MYA-4823). Thus, the genome sizes based on genome sequencing were larger than those based on the sum of chromosomal bands (Fig. 6B).

Densitometric analyses showed that both the ATCC MYA-4821 and ATCC MYA-4823 isolates had very high peak pixel intensities for the 7.0 Mb chromosomal band. The 7.0 Mb band was also strongly stained with EtBr (Figure S2A and Figure S3A, ATCC MYA-4821 and ATCC MYA-4823, respectively). In fact, the pixel intensities of the 7.0 Mb chromosomal band (113 for ATCC MYA-4821 *S. schenckii* and 80 for ATCC MYA-4823 *S. brasiliensis*,) were close to twice that of the 6.5 Mb chromosomal band (66 and 54 for ATCC MYA-4821 and ATCC MYA-4823, respectively; data not shown). This observation suggested the possibility that an additional chromosome may be present in the 7.0 Mb chromosomal band. This might explain the difference in genome size estimations discussed above. For example, when a second 7.0 Mb chromosome band was added to the sum of all chromosomal bands, the minimum genome sizes were 32.3 Mb and 32.7 Mb for ATCC MYA-4821 and ATCC MYA-4823, respectively. Therefore, our estimations of genome sizes would be very close to those reported based on genome sequencing. Taken together, our results strongly suggested that the 7.0 Mb chromosomal band might contain two or more chromosomes for the ATCC MYA-4823 and ATCC MYA-4821 isolates.

Another indication of chromosomal band co-migration arose from the *S. schenckii s. str.* gene mapping. The hybridization analysis suggested that the 6.5 Mb chromosomal band might harbor two co-migrating chromosomes. In most *S. schenckii* isolates, the probes Calm, ITS, CHS1, PKC, GProt, and Topo hybridized with the 6.5 Mb chromosomal band; however, in isolates Ss61 and Ss39, those probes hybridized with two chromosomal bands that ranged from 6.3 to 6.7 Mb (Figs. 4 and 5). The presence of a second 6.5 Mb chromosomal band would increase the average minimum genome size of the *S. schenckii* isolates to 32.0 Mb (Ss51, Ss137, Ss126, and Ss03) and 37.2 Mb (Ss141). Those values were very close to those reported from genome sequencing, except for the Ss141 isolate, where we estimated a size that was approximately 5.0 Mb higher than the size they estimated. This difference could be explained by karyotype polymorphisms that were previously described for this species.

## Discussion

Based on the phylogenetic analysis with sequences from Calm and ITS, we were able to differentiate among the species in the *S. schenckii* complex. In this study, we found tree topology that was similar to that reported in other studies that used only calmodulin sequences [3], [6], [9], [11], [32].

The electrophoretic karyotype profiles of *S. brasiliensis* isolates showed less variability than those observed in *S. schenckii s. str.* isolates. These results were consistent with the phylogenetic data [6], [9]; the variability among isolates within the species was less frequent in *S. brasiliensis* than in *S. schenckii s. str*. No correlation was found between the electrophoretic karyotype profiles and the cluster organizations among the isolates described in this study.

Variations in chromosome length are common in fungal strains, although the mechanism underlying chromosome length polymorphisms remains unclear. Potential mechanisms include a recombination between repeated sequences that are in the same orientation (which can cause deletions); chromosome breakage and repair (which can lead to translocations and/or deletions); heterochromosomal recombinations; recombinations between subtelomeric regions; and the loss of a chromosome that contains sequences that are not required for growth [40], [41]. Recently, chromosome fusion was observed in offspring of the fungus *Zymoseptoria tritici*, and the probable mechanism was attributed to a breakage-fusion-bridge cycle [42]. Chromosomal length polymorphisms are observed in both asexual and sexual fungi species. For example, in *Didymella rabiei*, the teleomorphic state of the phytopathogenic fungus *Ascochyta rabiei*, the karyotype polymorphism was observed among different species isolated across the globe; nevertheless, compared to asexual ascomycetes, *A. rabiei* had a relatively conserved karyotype [43]. This finding showed that sexual or asexual reproduction was independent of the degree of karyotype polymorphism within a species.

As expected, the present study revealed that the *S. schenckii* complex displayed extensive polymorphisms in chromosome number and size. The first report of polymorphisms in *S. schenckii s.l.* showed that isolates from a restricted area of Japan had 6 to 8 chromosomal bands, ranging from 0.5 to 6.2 Mb [18]. In the present study, we found no chromosomal bands smaller than 1.2 Mb; however, we identified chromosomal bands larger than 6.2 Mb. Most of the chromosomal bands observed in both studies were similar in size; however, in our study, polymorphisms in the number and sizes of chromosomal bands were evident. In the present study, we found that the *S. schenckii s. str.* isolate from Japan had 6 chromosomal bands, and the band sizes were similar to those found by Tateishi et al. [18]. These results showed that, despite different methodologies, our estimations of chromosomal bands sizes agreed with those reported previously. This agreement reinforced our finding that the *Sporothrix schenckii* complex had a high level of chromosome polymorphisms.

The electrophoretic karyotype profiles of isolates showed no correlation with the clinical characteristics or the sources of isolation. This was also described when other techniques were used, such as RAPD. In this study no correlation could be observed among the Brazilian isolates of *S. schenckii s.l.* from different geographic regions or among the clinical characteristics of sporotrichosis [44]. In *Paracoccidioides brasiliensis*, Feitosa et al. [21] compared isolates from different sources (human, animal, and soil), from different geographical regions (Brazil, Argentina, Colombia, and Peru), and with different clinical characteristics. They found no correlation between karyotype profiles and clinical/epidemiological characteristics. Canteros et al. [45] also found no correlation between the electrophoretic karyotype and geographic region in *Histoplasma capsulatum* isolated from Latin American patients. Thus, our results were consistent with those on other thermodimorphic fungi. The finding that chromosome rearrangements occurred suggested that they may play an important role in the structure of the genome.

Our results showed that the organization of the *Sporothrix* genome was relatively conserved among different isolates. The variations observed among the isolates suggested the occurrence of chromosome rearrangements, such as translocations. Some of the chromosome rearrangements and/or duplications observed in our study may also have occurred in *P. brasiliensis* [21]. The hybridization patterns observed with different probes suggested the presence of gene linkage groups and synteny among the *Sporothrix* species analyzed. The synteny was conserved in closely related species, consistent with observations in other fungi, like *P. brasiliensis*, where genes were linked on the same chromosome among different isolates [21], [46].

The finding that the 7.0 Mb chromosomal band from isolate Ss126 hybridized to multiple bands from the Brazilian isolates suggested the presence of repeated sequences in *S. schenckii s. str.* and *S. brasiliensis* isolates. The repeated sequences might reflect the chromosomal rearrangements observed in this study, because repeated sequences can provide a substrate for recombination, duplication, deletion, and inversion events [47]. It is interesting to note that, in *S. brasiliensis*, the strongest signals were observed in the small-sized bands, and the opposite occurred in *S. schenckii s. str*. Transposons (one class of repeated sequences) constituted 8–9% of the *P. brasiliensis* genome and 16% of the *P. lutzii* genome. Transposable elements were responsible for the addition of 3.0 Mb to the genome of *P. lutzii* [46]. These results suggested that the differences in chromosome sizes among *S. schenckii* complex isolates may be related to the number of repetitive sequences.

The minimum haploid genome size was calculated by summing the sizes of the chromosomal bands. For *S. schenckii s. str.*, we calculated an average minimum size of 29.3 Mb, similar to the 28 Mb reported by Tateishi et al. [18]. Torres-Guerrero [39] estimated a genome size of 45 Mb for *S. schenckii s.l.* isolates, based on the diphenylamine method. The discrepancy among these values may be related to the different isolates studied, because they can present chromosomal length polymorphisms, as well as to the different methodologies employed. The ploidy of *S. schenckii s. str.* remains uncertain. Torres-Guerrero found evidence for a diploid genome; however, the possibility of aneuploidy could not be rejected. Based on chromosome-size summations among *S. schenckii s. str.* isolates, genome size estimations have varied from 21.7 to 37.4 Mb; but these estimates were at least 2.07 fold lower than the genome size estimation reported by Torres-Guerrero [39].

The haploid genome sizes of *S. schenckii* (ATCC MYA-4821) and *S. brasiliensis* (ATCC MYA-4823), estimated from DNA sequencing, were 32.5 Mb and 33.4 Mb, respectively (Marcus de Melo Teixeira and Maria Sueli Soares Felipe, personal communication). Our results strongly suggested that the chromosomal band of 7.0 Mb, and in some cases, the band of 6.5 Mb, may have been duplicated in several isolates. The fluorescence intensities of these bands provided clues that suggested the presence of a duplicated band in isolates ATCC MYA-4821 and ATCC MYA-4823. When the duplicated chromosome was added to the sum of chromosomal bands, the calculated genome size was very close to those reported by groups that had assembled the genomes of *S. schenckii s. str.* and *S. brasiliensis*.

When genome sizes calculated by summing the chromosomal bands were compared to genome sizes obtained in genome sequencing, they generally tended to show high similarity. For instance, in *Microsporum canis*, this type of comparison suggested a doublet band that contributed to the genome size [48]. In contrast, for *Paracoccidioides brasiliensis* the genome size estimated by sequencing was similar to that estimated by PFGE [46].

*S. schenckii* complex isolates have shown variable levels of virulence, depending on the species [12], [14]. Generally, the most virulent species in the *S. schenckii* complex is *S. brasiliensis*, which showed a high degree of fungal burden in organs and was associated with high mortality [12], [14]. A previous study of these isolates revealed that they present variable degrees of virulence [14]. For instance, CBS 120339 and IPEC 16919 (*S. brasiliensis*) exhibited significantly higher fungal burdens and remarkable brain tropism compared to *S. schenckii s. str.* [12]. The rare species *S. luriei* (CBS 937.72) showed high virulence profile in murine model [13]. On the other hand, *S. mexicana* isolates (including CBS 120341) displayed little or no virulence in a murine model [12]. *S. schenckii s. str.* isolated from distinct regions of Brazil showed various degrees of virulence in the murine model. The *S. schenckii* were classified as: non-virulent (Ss143 and Ss39), low virulence (Ss141 and Ss16), medium virulence (Ss51 and Ss126) and high virulence (Ss40) [14]. Interestingly, despite the fact that isolate Ss39 (*S. schenckii*) was considered non-virulent, it was isolated from a human patient. One potential explanation for the disease was the presence of an underlying disease that compromised the host immune system [14].

A comparison between karyotype and virulence profiles showed that isolates with different degrees of virulence sometimes displayed similar karyotypes. For example, the extremely virulent *S. brasiliensis* isolates, CBS 120339 and IPEC 16919 [12], the highly virulent, *S. schenckii* isolate, Ss126, and the moderately virulent isolate, Ss51 [14], all had a similar profile of 3 large bands and 2 small bands. In contrast, the *S. schenckii* isolate with low virulence (Ss141) had 3 large bands, 2 intermediate bands, and one small band. Isolates with no virulence had different chromosomal band profiles. For example, Ss39 (*S. schenckii*) had 7 bands (4 large bands, 1 intermediate band, and 2 small bands) and isolate CBS 120341 (*S. mexicana*) had only four chromosomes (2 large bands and 2 intermediate bands).

Small chromosomes that carried virulence factor genes were described in *Alternaria alternata*, a phytopathogenic fungus. Strains of *A. alternata* pathotypes showed small chromosomes (<1.7 Mb), but these were not observed in nonpathogenic isolates [49]. The small chromosomes that we observed varied from 1.2 Mb to 3.5 Mb. Our results suggested that small chromosomes might carry virulence factors, because the nonpathogenic species, *S. mexicana* (CBS 120341) did not harbor any small chromosomes, and it was considered non/slightly virulent in other studies [12]. However, the mechanism of pathogenicity appears to be more complicated than simply the presence or absence of small chromosomes as evidenced by the isolate, Ss39, which carried small chromosomes, but the virulence level was distinct from the other isolates in a murine model.

The test for susceptibility to antifungal drugs [15] showed that *S. schenckii s. str.* and *S. brasiliensis* were highly susceptible to most antifungals tested *in vitro*; however terbinafine, ketoconazole, and posaconazole were the most effective drugs. On the other hand, *S. globosa* and *S. mexicana* were drug-resistant [15]. In *Candida albicans*, resistance to fluconazole was associated with chromosome loss and/or gain. *In vitro* experiments showed that aneuploidy could occur after a week of exposure to fluconazole [50], [51]. Also, in *C. neoformans*, an adaptive mechanism of resistance against azoles, called “heteroresistance”, was found to be due to azole-associated acquisition of aneuploidy after undergoing azole maintenance therapy [52]. It is unknown whether this mechanism might play some role in members of the *Sporothrix schenckii* complex, but isolates tested in previous studies were resistant to fluconazole [15]. Our results suggested that aneuploidy could occur in *Sporothrix*, and it is tempting to hypothesize that this phenomenon could be responsible for the differences in drug resistance, as described for other fungi [52]. Genome-wide mapping of fungal chromosomal bands would be a valuable future project that could elucidate the role of chromosomal polymorphisms in the biology of the *S. schenckii* complex.

## Conclusions

In light of recent taxonomic changes in the genus *Sporothrix* this study was the first to analyze chromosomal polymorphisms in diverse isolates of the *S. schenckii* complex and to assign genes to chromosomal bands of *S. schenckii s. str.* and *S. brasiliensis* isolated from Brazil. The minimum genome size of *S. schenckii* complex isolates was variable. Based on data from genome sequencing, we hypothesized that the isolates studied here may have harbored two 7.0 Mb chromosomes. The virulence in *Sporothrix* spp. is a complex phenomenon that may be orchestrated by polymorphisms in chromosomes, and deserves to be studied further. Overall, our results provided the number of chromosomes in these fungi; this information will facilitate the complete assembly of the genome sequences, and it will contribute to the understanding of the genomic organization of these species.

## Supporting Information

Figure S1 **Densitometric analysis of** ***S. schenckii*** **isolates from Brazil.** Each panel shows (*left*) the ethidium bromide-stained gel after pulsed field gel electrophoresis of chromosomes from the fungus strain indicated, and (right) a graph of the densitometric analysis. The size of each chromosomal band (Mb) is indicated on the left and above the corresponding peaks on the graph. Open arrows indicate where samples were loaded.
(TIF)

Figure S2 **Densitometric analysis of** ***S. schenckii*** **from the American continent and Japan.** Chromosomes are shown for fungi isolates from A: United States; B and D: Peru; C, E and G: Mexico; and F: Japan. Each panel shows (*left*) the ethidium bromide-stained gel after pulsed field gel electrophoresis of chromosomes from the fungus strain indicated, and (*right*) a graph of the densitometric analysis. The size of each chromosomal band (Mb) is indicated on the left and above the corresponding peaks on the graph. Open arrows indicate where samples were loaded.
(TIF)

Figure S3 **Densitometric analysis of** ***S. brasiliensis*** **from Brazil.** Each panel shows (*left*) the ethidium bromide-stained gel after pulsed field gel electrophoresis of chromosomes from the fungus strain indicated, and (*right*) a graphic of the densitometric analysis. The size of each chromosomal band (Mb) is indicated on the left and above the corresponding peaks on the graph. Open arrows indicate where samples were loaded.
(TIF)

Table S1 **Genes, primer sequences, PCR programs, and references used in this study.**
(DOCX)
