# Supplementary figures and images for: Chromosomal Polymorphism in the Sporothrix schenckii Complex
Source: PLoS One. 2014 Jan 23;9(1):e86819. doi: 10.1371/journal.pone.0086819 (PMC3900657; doi:10.1371/journal.pone.0086819)

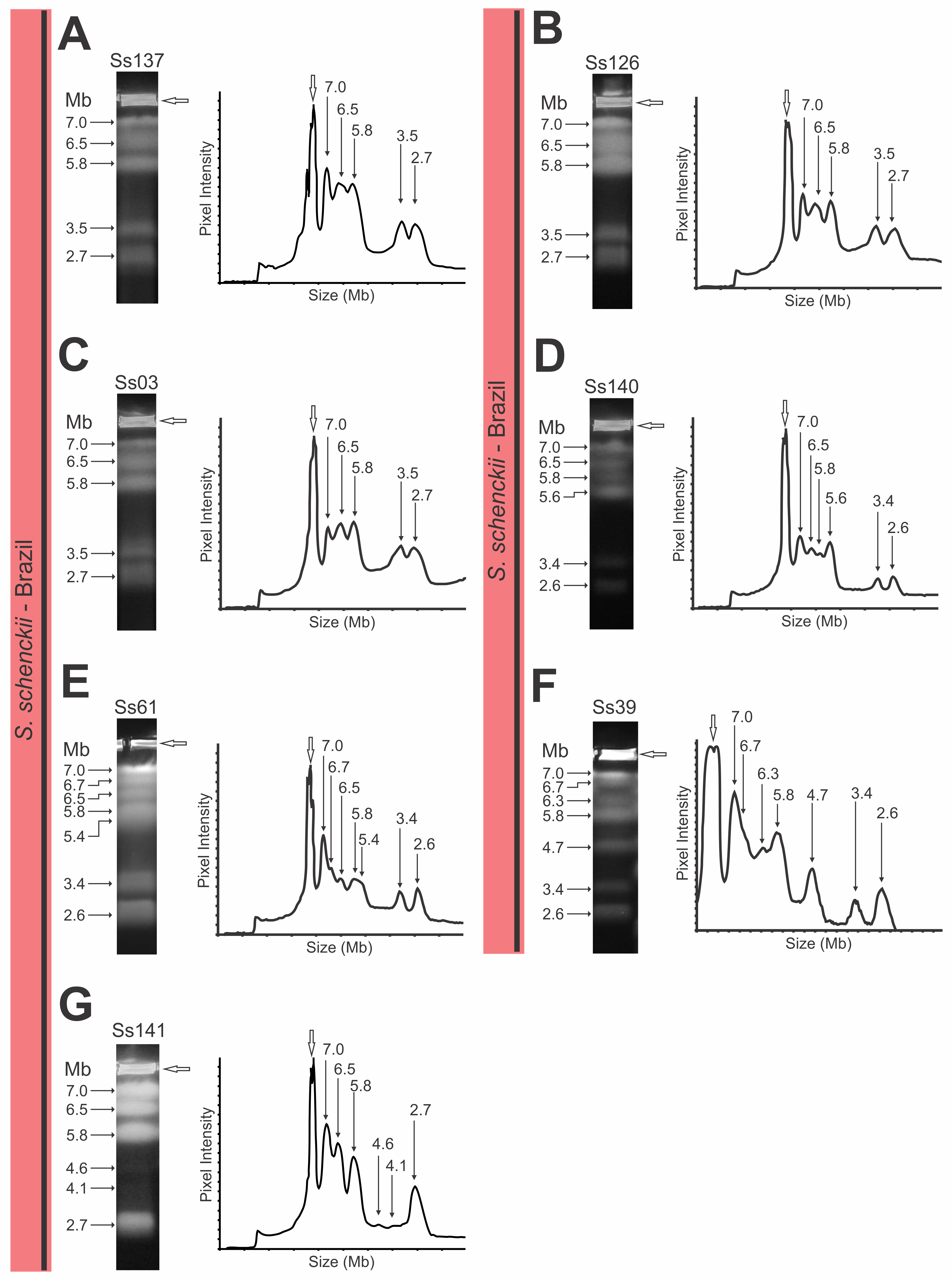

Supplement: Figure S1 — Densitometric analysis of S. schenckii isolates from Brazil. Each panel shows (left) the ethidium bromide-stained gel after pulsed field gel electrophoresis of chromosomes from the fungus strain indicated, and (right) a graph of the densitometric analysis. The size of each chromosomal band (Mb) is indicated on the left and above the corresponding peaks on the graph. Open arrows indicate where samples were loaded. (TIF) [file pone.0086819.s001.tif]

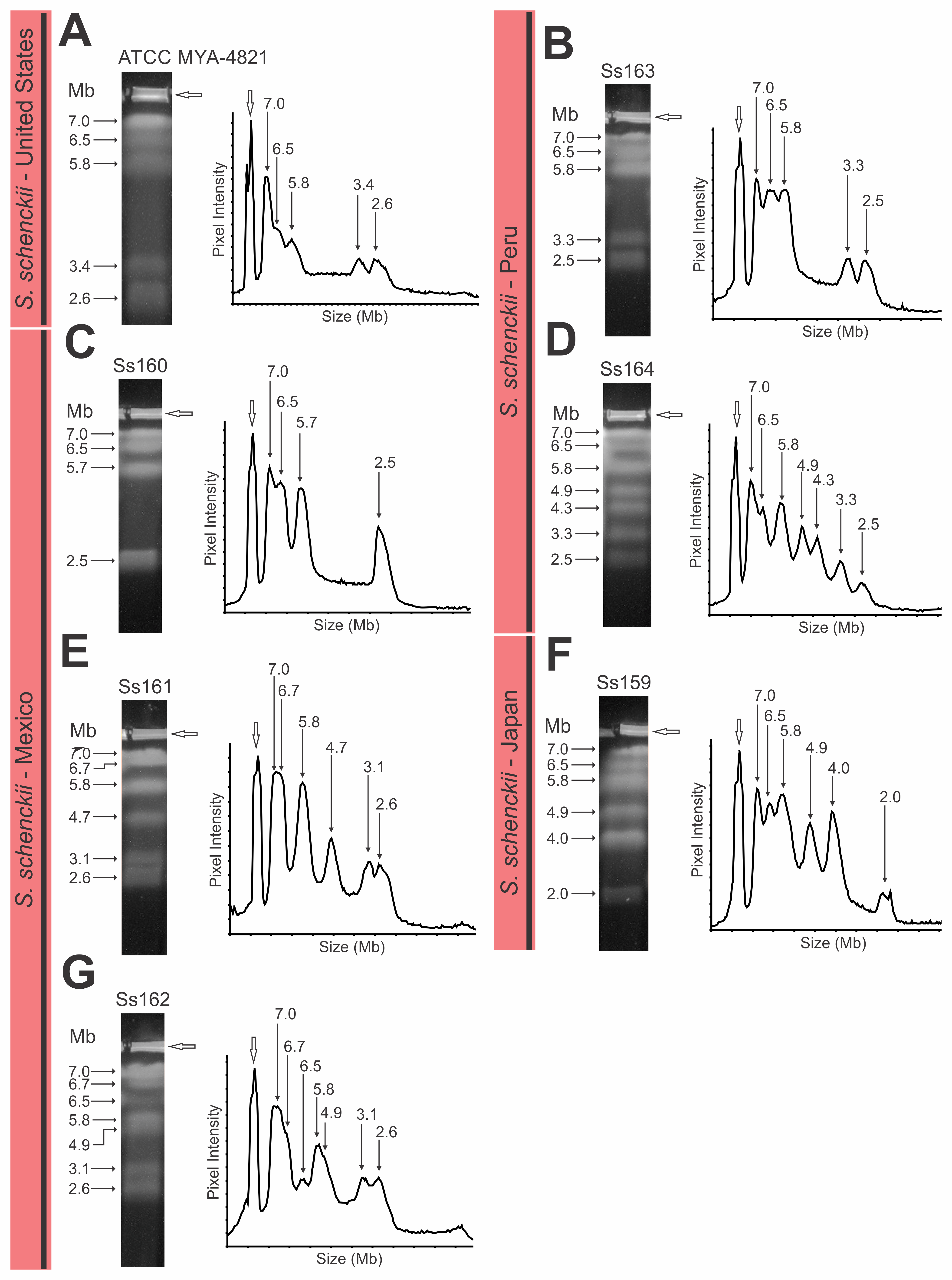

Supplement: Figure S2 — Densitometric analysis of S. schenckii from the American continent and Japan. Chromosomes are shown for fungi isolates from A: United States; B and D: Peru; C, E and G: Mexico; and F: Japan. Each panel shows (left) the ethidium bromide-stained gel after pulsed field gel electrophoresis of chromosomes from the fungus strain indicated, and (right) a graph of the densitometric analysis. The size of each chromosomal band (Mb) is indicated on the left and above the corresponding peaks on the graph. Open arrows indicate where samples were loaded. (TIF) [file pone.0086819.s002.tif]

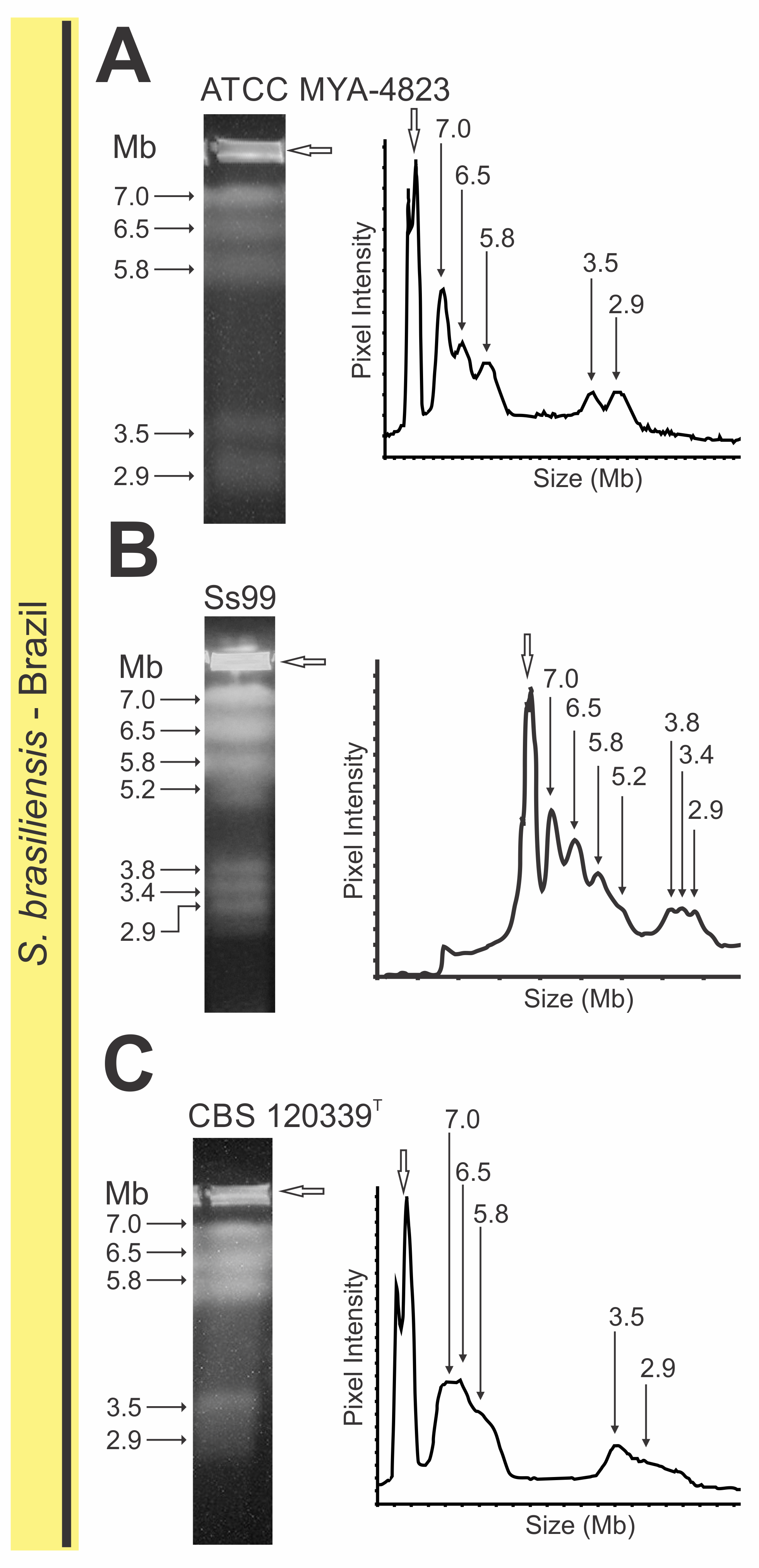

Supplement: Figure S3 — Densitometric analysis of S. brasiliensis from Brazil. Each panel shows (left) the ethidium bromide-stained gel after pulsed field gel electrophoresis of chromosomes from the fungus strain indicated, and (right) a graphic of the densitometric analysis. The size of each chromosomal band (Mb) is indicated on the left and above the corresponding peaks on the graph. Open arrows indicate where samples were loaded. (TIF) [file pone.0086819.s003.tif]
