# Supplementary material for: Chromosomal Polymorphism in the Sporothrix schenckii Complex
Source: PLoS One. 2014 Jan 23;9(1):e86819. doi: 10.1371/journal.pone.0086819 (PMC3900657; doi:10.1371/journal.pone.0086819)
Supplement: Table S1 — Genes, primer sequences, PCR programs, and references used in this study. (DOCX) [file pone.0086819.s004.docx]

| **Gene** | **Primer 1** | **Primer 2** | **Primer Set** | **PCR Cycles** | **Reference** |
| --- | --- | --- | --- | --- | --- |
| β-Tubulin | 5’ GGY AAC CAR ATH GGT GCY GCY 3’ | 5’ ACC CTC RGT GTA GTG ACC GGC 3’ | BT2F/BT2R | 94°C/5 min; 30 cycles: 94°C/1 min, 57°C/1 min, 72°C/2 min; 72°C/7 min | 10 |
| Calmodulin | 5’ GAR TWC AAG GAG GCC TTC TC 3’ | 5’ TTT TTG CAT CAT GAG TTG GAC 3’ | CL1/CL2A | 94°C/5 min; 35 cycles: 94°C/1 min, 60°C/1 min, 72°C/4 min.; 72°C/7 min | 22 |
| Catalase | 5’ TTG GGA CTT CCA CGT CGG CAA TCT 3’ | 5’ AAC TTG GGA CCA ATT TCG CTG GTG 3’ | CataF/CataR | 94°C/5 min; 35 cycles: 94°C/1 min, 65°C/45 sec, 72°C/1 min; 72°C/7 min | This study |
| Chitin Synthase 1 | 5’ TGG GGC AAG ATG CTT GGA AGA AG 3’ | 5’ TGG AAG AAC CAT CTG TGA GAG TTG 3’ | CHS-79F/CHS-354R | 94°C/5 min; 35 cycles: 95°C/30 sec, 55°C/1 min, 72°C/1 min; 72°C/7 min | 24 |
| ITS Region | 5’ TCC GTA GGT GAA CCT TGC GG 3’ | 5’ TCC TCC GCT TAT TGA TAT GC 3’ | ITS1/ITS4 | 94°C/2 min; 35 cycles: 94°C/30 seg, 59°C/30 sec, 68°C/1 min; 68°C/7 min | 25 |
| Pho85 Cyclin-Dependent Kinase | 5’ ATG GAT GGC AAA CGC CAA CTG AAC TC 3’ | 5’ TCC CGC GGC TTG CTG GCA GTG CTG G 3’ | Pho85F/Pho85R | 94°C, 5 min; 35 cycles: 94°C/1 min, 65°C/45 sec, 72°C/1 min; 72°C/7 min | This study |
| Protein Kinase C Ss-2 | 5’ GCG GAA GAG CAG ATG CAA CAG 3’ | 5’ GGG CGC CAT AAA CTC GGG AGT 3’ | QuiCF/QuiCR | 94°C/5 min; 35 cycles: 94°C/1 min, 60°C/45 sec, 72°C/1 min; 72°C/7 min | This study |
| G Protein α Subunit | 5’ ATG GGT TGC GGA ATG AGT GTG GAG G 3’ | 5’ AAG ACC ACA TAG ACG CAA GTT CTC CT 3’ | GProtF/GProtR | 94°C/5 min; 35 cycles: 94°C/1 min, 65°C/45 sec, 72°C/1 min; 72°C/7 min | This study |
| Topoisomerase II | 5’ GCA GCC CAC GTC CAA CAA GAC T 3’ | 5’ GTC AGA GGT CTT ATT GGA CGT GA 3’ | SSHF31/SSHR97 | 94°C/5 min; 30 cycles: 94°C/1 min, 57°C/1 min, 72°C/2 min; 72°C/7 min | 29 |
